# Effect of the administration of probiotics on the fecal microbiota of adult individuals

**DOI:** 10.1002/fsn3.2547

**Published:** 2021-10-06

**Authors:** Feiyan Zhao, Hao Jin, Xin Shen, Qi Li, Xiaoye Liu, Lei Zhang, Zhihong Sun, Jie Yu

**Affiliations:** ^1^ Key Laboratory of Dairy Biotechnology and Engineering Key Laboratory of Dairy Products Processing Inner Mongolia Agricultural University Hohhot China; ^2^ 117454 Inner Mongolia Key Laboratory of Dairy Biotechnology and Engineering Inner Mongolia Agricultural University Hohhot China

**Keywords:** enterotype, gut microbiota, healthy adults, probiotic

## Abstract

Probiotics have been used to ameliorate ailments by modulating gut microbiota. However, to date, the effects of probiotic supplementation on the composition of fecal microbiota in healthy adults remain obscure. In this study, nine healthy volunteers were recruited to take probiotics (a mixture of *Lactobacillus casei* Zhang, *L*. *plantarum* P‐8, and *Bifidobacterium lactis* V9, 2:2:3, 1 × 10^10^ CFU/day) for 28 days. The fecal samples were collected at 0 and 28 days, and V4 of the 16S rRNA gene sequenced by Illumina MiSeq was used to analyze the fecal microbiota. The enterotype has been used to characterize the composition of gut microbiota. Nine adults were divided into Type P (fecal microbiota dominated by *Prevotella*, 4 adults) and Type B (fecal microbiota dominated by *Bacteroides*, 5 adults) based on an enterotype analysis. The responses of variation had been found in two enterotypes. The α‐diversity was not changed significantly after the administration of probiotics in both Type P and B. However, the community structure in Type B was substantially influenced. After the administration of probiotics, *Weissella* and *Leuconostoc* were significantly higher in Type P, while *Collinsella* significantly increased in Type B. The different pathways involving pathogen infections were downregulated at 28 days. The Type VI secretion system and the EHEC/EPEC pathogenicity signature were downregulated in Type B and Type P, respectively.

## Graphical abstract

The response to probiotics varies among populations with different enterotypes, but probiotics can improve the health of different enterotypes by modulating gut microbiota and its functional pathway in different ways.

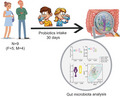

## 1 INTRODUCTION

The human gut microbiota refers to the microbes that reside inside the gut and participate in several functions that are beneficial to the host, including the fermentation of indigestible dietary fibers, the prevention of pathogen colonization, and the maturation and regulation of the immune system (Paone & Cani, 2020). The emergence and development of high‐throughput sequencing techniques in recent years have deepened our understanding of the role of microbiota in health and illness. It has been elucidated that several clinical diseases, such as colorectal cancer (Song et al., 2020), rheumatoid arthritis (Chiang et al., 2019), and neurological disorders (Cryan et al., 2020), occur owing to the dysbiosis of intestinal flora. Thus, the role of gut microbiota as potential risk factors for the onset of disease development has been the subject of intensive study.

The composition and structure of gut microbiota are closely related to diet, genes, age, and drug. Although the gut microbiota is immensely separated between individuals, the concept of “enterotypes” has been proposed to improve the understanding of the role that gut microbiome play in health. According to the dominant bacteria in the intestinal tract, the gut microbial community structures of adult human beings are classified into three types, and each type is defined by the high abundance of *Bacteroides*, *Prevotella*, and *Ruminococcus* (Costea et al., 2020). Chung et al. reported that the amount of arabinoxylan‐oligosaccharides can significantly increase the abundance of both *Bifidobacterium* and *Prevotella* in *Prevotella*‐plus volunteers, while only *Bifidobacterium* changed in the samples of *Prevotella*‐minus individuals, which indicated the different responses to food based on the characteristics of gut microbiota (Chung et al., 2020).

Probiotics are defined as “live microorganisms which can confer a health benefit on the host when administered in adequate amounts” (McFarland, 2015). Based on the population of specific pathologies, probiotics have been proven to benefit the gut environment and the health of host by regulating the balance of gut microbes and improving the resistance of host to pathogenic bacteria (Xu et al., 2017, 2019). However, there is limited and less consistent evidence that shows the health‐promoting effect of probiotics on healthy participants (Hou et al., 2020; Lee et al., 2020; Wang et al., 2015), which is related to the probiotic strains, ingestion time, viable count, and formulations of probiotic products. Probio‐Fit^®^, consisting of *L. casei* Zhang, *L. plantarum* P‐8, and *B. lactis* V9 (2:2:3), has been shown to have a favorable curative effect in the adjuvant treatment of IBS (Chen et al., 2020) and IBD patients, but its effect on healthy individuals remains unclear. Therefore, this study was designed to investigate changes in the composition and diversity of gut microbiota caused by supplementation with Probio‐Fit^®^ based on enterotypes (Table 1).

**TABLE 1:** Information of nine volunteers

Number	Age	Gender	Ethnicity	BMI	Bristol Scores	Residence
	(years)			(kg/m^2^)		
A1	31	Female	Han	21	3	Shanghai, China
A2	35	Female	Han	23	3	Shanghai, China
A3	36	Female	Han	29	4	Shanghai, China
A4	29	Male	Han	24	3	Shanghai, China
A5	30	Female	Han	24	4	Shanghai, China
A6	33	Male	Han	25	3	Shanghai, China
A7	38	Male	Han	29	4	Shanghai, China
A8	27	Male	Han	25	3	Shanghai, China
A9	31	Female	Han	23	3	Shanghai, China

## 2 MATERIAL AND METHODS

### 2.1 Study participants

Nine participants (five males and four females) were randomly recruited from the same physical examination center of Shanghai, China, when they were examined. All the subjects were between 27 and 38 years old, with a body mass index (BMI) < 30 kg/m^2^. The subjects had no endocrine disorders, diabetes, or gastrointestinal problems and had not taken antibiotics for six months before and during the study period. The participants ceased consuming alcoholic beverages and other probiotic products.

### 2.2 Experimental design

The probiotic powder Probio‐Fit^®^ containing a 1.0 × 10^10^ CFU mix of probiotics was packed in individually sealed sachets and stored at 4℃ before consumption. All the participants were required to take the probiotics for 28 days. Probio‐Fit^®^ was consumed in warm water half an hour after lunch each day and provided by the Beijing Scitop Bio‐tech Shareholding Co. Ltd.

The fecal samples of all the subjects were obtained at day 0 (before the supplementation) and day 28 (post supplementation). The sampling process enabled adequate fecal sampling without contamination. All the samples were transported to the laboratory in dry ice and stored at −80°C until further processing.

### 2.3 DNA extraction and sequencing

The genomic DNA extraction of fecal samples was performed using a QIAGEN DNA Stool Fast Kit (QIAGEN) following the manufacturer's instructions. The quality of the extracted metagenomic DNA was checked using 1.0% agarose gel electrophoresis and a spectrophotometric analysis (optical density at a ratio of 260 nm/280 nm). The concentration of DNA was required to be >20 ng/μl, and the 260 nm/280 nm ratio was required to be 1.8‐2.0.

The extracted DNA was used as the template for amplification of the V4 region of the 16S rRNA gene with a specific barcode primer set. The primers contained Illumina adapters, and the reverse primer contained a barcode sequence unique to each sample. High‐throughput sequencing of the PCR products was performed using an Illumina MiSeq platform (MiSeq PE250; Illumina) at PROMEGENE.

### 2.4 Bioinformatics analysis

The V4 region of the 16S rRNA gene of all the samples was sequenced. All the DNA sequences were spliced and divided into samples according to the nucleotide tag (barcode) information, and the barcode and primers were excised to obtain high‐quality sequences. Quantitative Insights into Microbial Ecology (QIIME) package (version 1.7) (Caporaso, Bittinger, et al., 2010) was then used to conduct a bioinformatics analysis on the extracted high‐quality sequences. The main processing flow was as follows: (1) PyNAST (Caporaso, Kuczynski, et al., 2010) was used for sequence calibration and alignment; (2) a single sequence set was constructed according to 100% based on UCLUST (Edgar, 2010); (3) the unique sequence set was classified into operational taxonomic units (OTUs) under the threshold of 97% identity using UCLUST after the selection of the representative sequences; (4) Chimera Slayer (Haas et al., 2011) was adopted to remove the OTUs that contained chimeric sequences; (5) representative sequences of each OTU without a chimera were selected and compared with the sequence homology with RDP (Ribosomal database project, Release 11.5) (Cole et al., 2007), Green Genes (Release 13.8) (DeSantis et al., 2006), and Sliva (Version 132) to assign their taxonomic level of phylum, class, order, family, and genus (Quast et al., 2013); (6) Shannon‐Wiener and Simpson diversity were computed on the basis of the de novo taxonomic tree constructed by the representative chimera‐checked OTU set using FastTree (Price et al., 2009). Shannon–Wiener and Simpson diversity analyses were performed to evaluate the depth of sequence and biodiversity richness of the samples; (7) Bray–Curtis distances were calculated, and Nonmetric Multi‐dimensional scaling (NMDS) based on the Bray–Curtis distances were applied to assess the β‐diversity analysis of the microbiota structure of different samples. Linear discriminant analysis effect size (LEfSe) was then performed to find the biomarker before and after the probiotics using the default parameters. The bacterial diversity was predicted from 16S rRNA gene‐based microbial compositions, and functional inferences were made from the Kyoto Encyclopedia of Gene and Genomes (KEGG) catalog using the PICRUst algorithm.

### 2.5 Sequencing data accession numbers

The raw sequence data in our study were deposited in the Metagenome Rapid Annotation using Subsystem Technology (MG‐RAST) database (accession number mgp94006, https://www.mg‐rast.org/linkin.cgi?project=mgp94006).

### 2.6 Statistical analysis

Statistical analyses were performed using R packages (http://www.r‐project.org/). Based on the genus abundance profile, enterotype analysis was performed as described and visualized by the R “ade4” package. The intragroup differences in the two enterotypes were compared using a Wilcoxon test, and *p*‐values <.05 were considered statistically significant. Linear discriminant analysis (LDA) scores of LEfSe >2 were considered to be significant. The annotated sequences were also assigned to the KEGG orthologue group (KO) according to the highest score, and Z‐scores were calculated to express the differences in enriched metabolic pathways before and after the probiotics. Z‐scores >1.6 were considered to be statistically enriched. Finally, the analysis results were visualized by R packages, Origin 8.6 (OriginLab) and GraphPad 7.0 (GraphPad Prism).

## 3 RESULTS

### 3.1 Sequencing coverage analysis and enterotype‐like clusters

**FIGURE 1:**
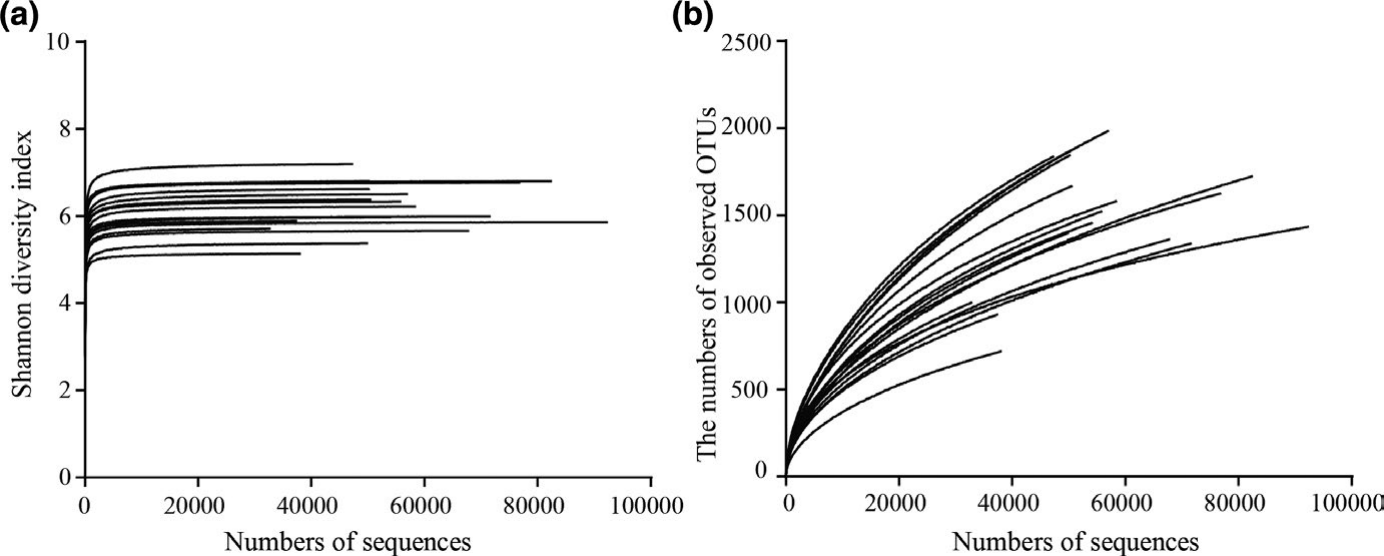
Shannon diversity index curves (a) and Rarefaction curves (b) for all fecal samples. OTU: operational taxonomic units

To characterize the effects of probiotics on the intestinal microbiota of healthy adults, V4 of the 16S rRNA genes was sequenced using the Illumina MiSeq platform. A rarefaction model was used to evaluate the species richness of the sampling data (Figure 1). It was determined that current sequencing had represented most of the microbial diversity. However, more phylotypes could be found by increasing the depth of sequence.

**FIGURE 2:**
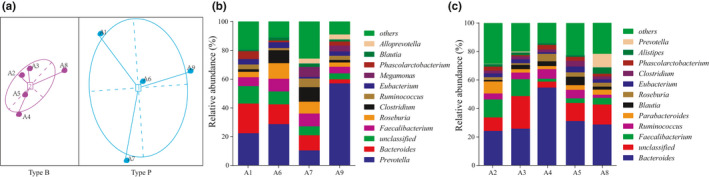
The enterotype analysis based on the fecal microbiota of volunteers at day 0 and the relative abundance of bacteria at the genus level of two enterotypes (more than 1.00% of all sequences). (a): visualization by principal coordinate analysis, (b): Type P, (c): Type B

We analyzed the bacterial microbiota composition of all the samples (a total of 18, from two different time points). The fecal microbiota at 0 day were analyzed adopting the method described by Arumugam M (Arumugam et al., 2011), and the results showed that the fecal microbiota fell into two distinct enterotypes described in Figure 2a Type P (four individuals) was dominated by *Prevotella* (contributing 29.51% of the total sequence), while Type B (five individuals) was characterized by the dominance of *Bacteroides* (contributing 32.81% of the total sequence) as shown in Figure 2b,c.

**FIGURE 3:**
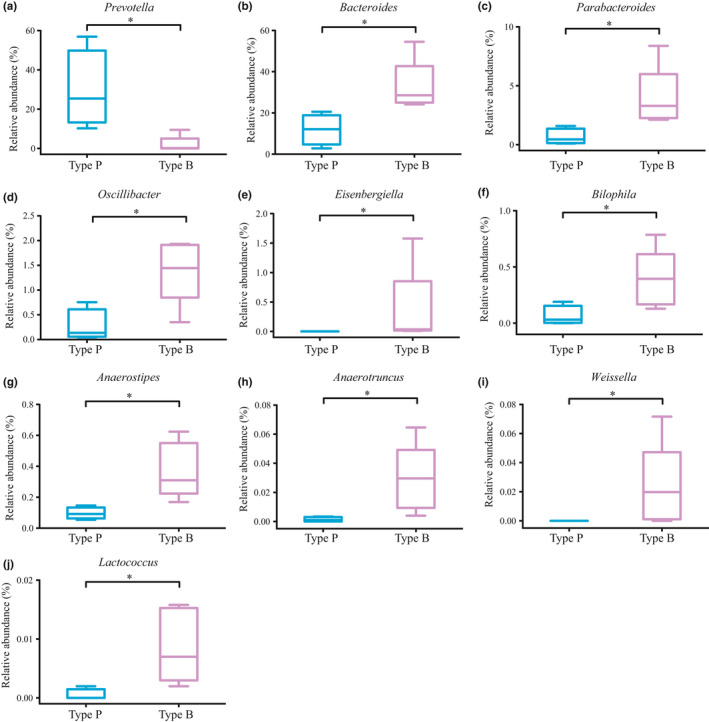
Different relative abundance of bacteria at the genus level of two enterotypes. Significant correlations are represented by *:.01 < *p* < .05

At the phylum level, more than 99.07% of the sequences belonged to the four most populated bacterial phyla in Type P and Type B, namely Firmicutes, Bacteroidetes, Proteobacteria, and Actinobacteria (48.23% vs 47.46%, 45.5% vs 44.47%, 3.4% vs 4.46%, and 2.08% vs 2.68%, respectively). At the genus level, 14 and 19 genera were the dominant bacterial genera in Type P and Type B, respectively (relative abundance of >1%). Interestingly, *Prevotella* was the most abundant genus in Type P, while it was less abundant in Type B. Moreover, 10 bacterial genera were observed to differ significantly between these two enterotypes. The proportion of *Bacteroides*, *Parabacteroides*, *Oscillibacter*, *Anaerostipes*, *Anaerotruncus*, *Bilophila*, *Eisenbergiella*, *Lactococcus,* and *Weissella* (*p* < .05) in the Type B subgroup was significantly higher compared with those in Type P, while *Prevotella* was significantly abundant in the Type P subgroup (Figure 3).

### 3.2 Effect of probiotics on the gut microbial α‐diversity and β‐diversity

**FIGURE 4:**
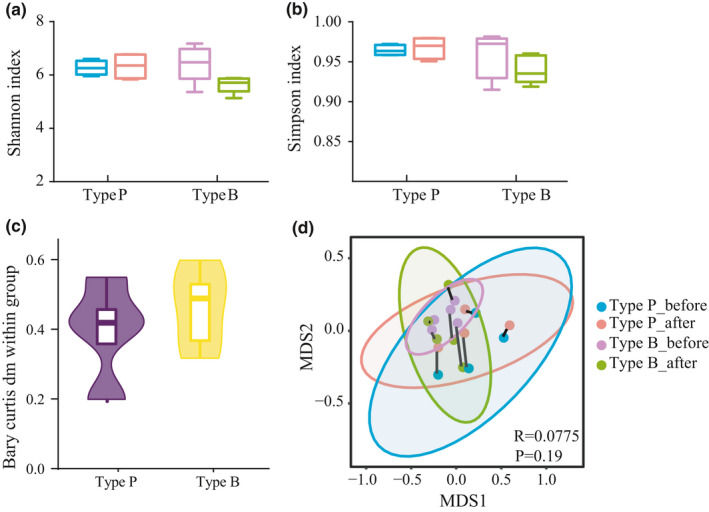
α‐diversity and β‐diversity analyses on fecal microbiota before and after the intervention of probiotics. (a): The change in Shannon index after the administration of probiotics. (b): The change in Simpson index after the administration of probiotics. (c): NMDS analysis plots based on the Bray–Curtis distances. (d): The variation of individuals based on the Bray–Curtis distance)

The Shannon and Simpson indices were used to evaluate the change of α‐diversity before and after the probiotics were consumed. The Shannon index (Figure 4a) and Simpson index (Figure 4b) changed slightly after the administration, but no significant difference was observed (*p* > .05).

The NMDS analysis based on the Bray–Curtis distances revealed bacterial structural differences between the two different enterotypes in both Type P and Type B, but no clear clusters were observed before and after the intervention of probiotics (Figure 4c). However, it is noteworthy that the samples that belonged to the same individual mostly clustered together (connected by lines on the plot), suggesting that the individual difference might be greater than that of the probiotic effects on the fecal microbiota structure. Therefore, the variable difference in the Bray–Curtis distances based on individuals before and after ingestion was calculated to decrease the difference between participants. The results showed the variation of Type B was higher than that of Type P (Figure 4d), which indicates that the structure of Type B could be more disturbed than that of Type P. However, no statistically significant difference was observed.

### 3.3 Probiotics‐directed changes in the composition of gut microbiota

To determine the change of microbiota composition after the probiotics, the LEfSe analysis was used to identify differences in the taxa before and after the administration in the two enterotypes.

**FIGURE 5:**
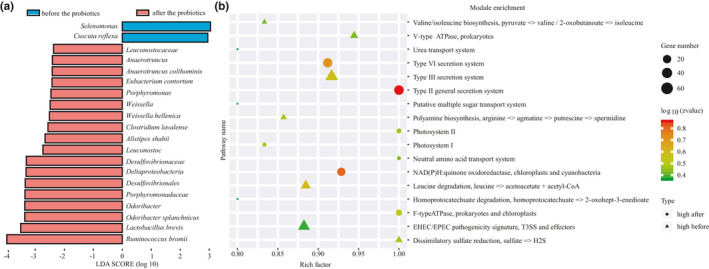
Identification of bacterial biomarkers (a) by LEfSe analysis and KOs change (b) of volunteers in Type P before and after the probiotics. LDA: Linear discriminant analysis, the LDA scores >2 considered as significant changes

**FIGURE 6:**
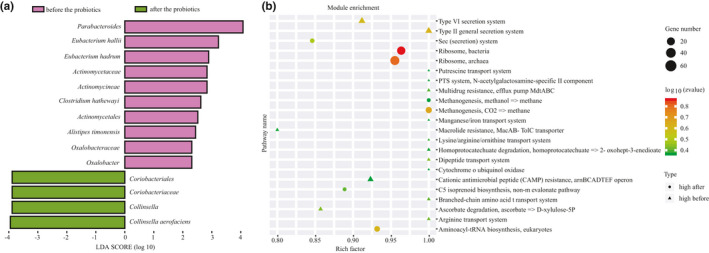
Identification of bacterial biomarkers (a) by LEfSe analysis and KOs change (b) of volunteers in Type B before and after the probiotics

Varied responses were found between the two types. In Type P, 20 taxa of different taxonomic levels were found before and after the administration of probiotics (Figure 5a, LDA Score >2). The relative abundances of *Anaerotruncus*, *Desulfovibrionales*, *Porphyromonas*, *Weissella,* and *Leuconostoc* were significantly higher, while the abundance of *Selenomonas* decreased significantly. Whereas only 14 different taxonomic levels were found in the Type B group (Figure 6a, LDA Score >2), *Collinsella* is one of the genera in Coriobacteriaceae, which was significantly higher than before the probiotics. Moreover, the genera *Oxalobacter* and *Parabacteroides* decreased significantly after the consumption of probiotics. The varied responses in the two types may relate to their characteristics.

### 3.4 Changes in microbial metabolic pathways and functional features

To investigate the effects of administration of probiotics on the functional profiles, high‐quality reads from two different enterotype samples were assembled and annotated for their protein‐coding genes and generated for the KEGG database orthologues (Figure 5b and Figure 6b).

Varied responses were observed again. After the administration of probiotics, the function of two enterotypes clustered closer, which indicates that the same metabolic modules of individuals changed in the opposite direction after the administration of probiotics. For example, phosphate and amino acid transport systems decreased in Type P, but they increased in Type B after the probiotics. Some modules in carbohydrate and lipid metabolism and energy metabolism were higher in the subjects of Type B after the administration of probiotics, which were higher in the subjects of Type P before the administration of probiotics.

In addition, we also found some similarities in the two enterotypes, such as the significant downregulation of pathogenic bacteria‐related metabolic pathways after the administration of probiotics. In the Type P group, the KOs of Type III secretion system, polyamine biosynthesis, and EHEC/EPEC pathogenicity signature were significantly decreased, while the KOs of Type VI secretion system were significantly dropped in Type B.

## 4 DISCUSSION

In this study, the effects of the probiotic's administration on fecal microbiota in healthy individuals were investigated. The concept of “enterotype” was proposed by Arumugam M (Arumugam et al., 2011), and the individuals of different countries were categorized into three enterotypes based on the differences in general, namely *Bacteroides*, *Prevotella,* and *Ruminococcus*. In our study, the variation of gut microbiota in nine healthy individuals clustered into two enterotype‐like groups at the genus level driven by *Prevotella* (Type P) and *Bacteroides* (Type B), which were consistent with the findings of a meta‐analysis using the Human Microbiome Project (HMP) database (Koren et al., 2013). The enterotype stratifications were not specific to a nation or continent, which could reflect different responses toward diet intake (Wu et al., 2011). Enterotype P, driven by *Prevotella*, has been shown to be rich in individuals with fiber‐rich diets. Conversely, enterotype B represented by *Bacteroides* has been related to diets enriched in saturated fats and animal proteins (Costea et al., 2018). These variations among enterotypes reflect different combinations of microbial trophic chains with a probable impact on energy balance in the hosts (Christensen et al., 2018). Therefore, the different responses of the two enterotypes to drugs were analyzed in some studies. A study on the effects of Acarbose on type‐2 diabetic patients showed that patients with different enterotypes (Type B and Type P) showed different responses in metabolic parameters (Gu et al., 2017).

Such interesting results also occurred in this study, which showed various responses to probiotic administrations in different enterotypes. The α‐diversity did not change significantly in two enterotypes after the administration of probiotics, which is consistent with previous findings that the α‐diversity of intestinal flora was not significantly affected by the administration of probiotics (Noh et al., 2018). Examinations of the β‐diversity indicated that the samples that belonged to the same individual mostly clustered together regardless of the probiotic interventions. However, after removing individual differences, it appeared that Type B was more vulnerable than Type P when the probiotics were administered. The same observation has been obtained in a previous study (Haddad et al., 2020; Kang et al., 2016), which indicates that the ability of probiotic intervention to alter the gut bacterial community was affected by the baseline intestinal community. Additionally, a healthy flora must have the potential properties of resilience to internal factors (such as age) and external factors (such as dietary) perturbation and recover a healthy balance (Lloyd‐Price et al., 2016). Thus, our results showed that the fecal microbiota structure of the subjects changed modestly after the administration of probiotics.

This study further identified microbial biomarkers with LEfSe analysis to identify the change in composition of the microbiota induced by the administration of probiotics. Different responses to the probiotics in two enterotypes were observed again. In Type P, a substantial increase was observed in the abundances of *Anaerotruncus*, *Porphyromonas*, *Weissella,* and *Leuconostoc*, while the abundance of *Selenomonas* simultaneously decreased after the probiotics. *Weissella* and *Leuconostoc* have been recognized as beneficial for health. *Weissella cibaria JW15* has been proven to effectively enhance immune functions by increasing the cellular activity of NK (Lee et al., 2018). There are several strains of *Leuconostoc* that have proven to have probiotic characteristics, such as the production of butyric acid, the regulation of blood glucose, and improvement of metabolic dysfunction related to obesity (Castro‐Rodriguez et al., 2020; Traisaeng et al., 2020). Compared with healthy people, the prevalence of *Selenomonas* has been found to be significant in the gut of systemic lupus erythematosus patients (Graves et al., 2019). In Type B, an increase in *Collinsella,* a genus of Coriobacteriaceae was observed. Coriobacteriaceae species were reported to decrease in the gut of Crohn's patients in a systematic review (Pittayanon et al., 2020), and some species in this family possess the ability to convert bile acid. *Collinsella,* acting as the dominant taxon of Coriobacteriaceae, has a strong relationship with the circulation of insulin (Gomez‐Arango et al., 2018). Fructose‐lysine is a common Maillard reaction product that is harmful to health. Gomez et al., found that *C. intestinalis* and *C. aerofaciens* could transfer and catabolize ϵ‐fructose‐lysine into lysine, formic acid, and acetic acid in the intestinal tract (Wolf et al., 2019). Both butyric acid and acetic acid are short‐chain fatty acids, which show beneficial effects on health and may prevent the colonization of harmful bacteria. It could be inferred that the administration of probiotics could confer different effects on different subjects. However, those effects are likely to promote health.

The changes in bacterial diversity and population may lead to the regulation of metabolic pathways. Therefore, it is paramount to analyze the regulation of pathways and their functional features. Alterations in the microbial metabolic pathways were also observed in two enterotypes. The same metabolic modules of individuals were found to cluster in one enterotype after the administration of probiotics but dispersed in another, which could be related to the small number of samples. This is a limitation of this study and merits additional experiments based on a large population to substantiate it. Enteropathogenic *Escherichia coli* (EPEC) and enterohemorrhagic *Escherichia coli* (EHEC) are foodborne pathogens and can cause gastrointestinal diseases (Tack et al., 2020). However, the pathways of III secretion system and EHEC/EPEC pathogenicity signature are downregulated in Type P and Type VI, and the II general secretion system is downregulated in Type B after the administration of probiotics. Type II, III, and VI secretion systems are related with the infections of gram‐negative pathogens (Fox et al., 2020; Pinaud et al., 2018). Therefore, the low expression of EHEC/EPEC pathogenicity signature and Type II, III, and VI secretion systems after the administration of probiotics demonstrated their beneficial effect on health.

## 5 CONCLUSIONS

Our study demonstrated various responses owing to the administration of probiotics in different enterotypes, suggesting that probiotics are likely to colonize in the intestinal microbiota of an individual depending on its characteristics. However, the administration of probiotics is likely to promote the health of the individual in both Type P and Type B by enhancing the growth of beneficial bacteria and inhibiting the pathways related to pathogenesis.

## CONFLICTS OF INTEREST

There are no conflicts of interest to declare.

## AUTHOR CONTRIBUTIONS

**Feiyan Zhao:** Data curation (equal); Methodology (equal); Software (equal); Writing‐original draft (lead); Writing‐review & editing (equal). **Jie Yu:** Conceptualization (equal); Formal analysis (equal); Resources (equal); Writing‐review & editing (equal). **Jin Hao:** Data curation (equal); Methodology (equal); Software (equal); Validation (equal). **Xin Shen:** Investigation (equal); Methodology (equal); Project administration (equal); Visualization (equal). **Qi LI:** Formal analysis (equal); Investigation (equal); Visualization (equal). **Xiaoye Liu:** Methodology (equal); Project administration (equal); Supervision (equal). **Lei Zhang:** Investigation (equal); Project administration (equal); Visualization (equal). **Zhihong Sun:** Conceptualization (equal); Funding acquisition (equal); Resources (equal).

## ETHICS APPROVAL

All the study procedures were approved by the Ethical Committee of Inner Mongolia Agricultural University (Hohhot, China) and our study conforms to the Declaration of Helsinki, US, and European Medicines Agency Guidelines for human subjects. The informed consent of all patients involved in this study obtained and informed consent before the trail.
